# Building resources to meet evolving laboratory medicine challenges in Africa

**DOI:** 10.4102/ajlm.v7i1.915

**Published:** 2018-11-29

**Authors:** Iruka N. Okeke

**Affiliations:** 1 Department of Pharmaceutical Microbiology, University of Ibadan, Ibadan, Nigeria

## Building resources to meet evolving laboratory medicine challenges in Africa

Two brief reports in this issue spotlight intersections between communicable and non-communicable disease. Nabaigwa et al.^1^ report on urinary tract infections in diabetic patients at a Ugandan hospital, where they found hyperglycaemia associated with urinary tract infections, and Ashaka et al.^2^ describe anaemia of likely infectious origin: human parvovirus B19. As these reports illustrate, disease, as we know it, has become more complicated. Each patient will likely be best served by multiple tests performed in different laboratory medicine departments in order to get the most informative description of his or her disease. At no time before now has laboratory medicine been so central to healthcare delivery.^3^

Three decades ago, a yes or no response to the question: ‘Is this patient infected with HIV?’ might have been sufficient to initiate state-of-the-art treatment in Africa and was well above the standard of care.^4,5^ Since then, the use of CD4+ counts and HIV viral load information to guide care has come from gains in knowledge and the appreciation that appropriate diagnosis and treatment access is central to containing the disease.^6^ However, diagnostic needs have advanced even beyond that standard for reasons that are actually determined by the virus: HIV drug resistance is a new challenge, brought about by viral evolution and the selective pressure from antiretrovirals. Thus, new knowledge and disease evolution will continue to press us to change. In this issue, Zhou et al.’s^7^ response to the need for drug resistance information was to evaluate the application of viral population deep sequencing for people living with HIV in Malawi. They demonstrate that deep sequencing can identify drug-resistant minority variants, making informed drug choices that eliminate rather than select resistant HIV lineages possible. The question that their study raises, because the study used Roche 454 sequencing via a research collaboration, is how to apply strategies like it routinely at the point of care in Malawian and other African clinics. This is a ‘When?’ and not a ‘Whether?’ question of course: once next generation sequencing is available for diagnosis of one disease, it can be used for a range of diagnostics with very little additional infrastructural and human resource development.

Some of the technologies that will aid laboratory contributions to better care are relatively new ones, operating on new platforms and taking advantage of what we now know about the molecular basis of disease. Zhou et al.’s^7^ next generation sequencing approach is a case in point. But still others will be extensions of tried and true technologies that may not require new skills and infrastructure. For example, Zhang et al.^8^ (this issue) described the results of field testing a simple thin layer agar culture-based method that can detect drug-resistant tuberculosis in under 2 weeks using chromogenic plates that can be cultured in remote or resource-limited settings. While not as fast, they found it to be almost as accurate as Xpert^®^ MTB/RIF testing and much cheaper.

Both the Zhang^8^ and Zhou^7^ articles showcase techniques that have already been deployed in other parts of the world but had not previously been piloted in African healthcare settings. In the end, an evidence-based selected suite of essential phenotypic and genotypic tests, including sequence-based ones, must be available to all patients and the more rapidly we can assess test utility in local settings, the sooner African patients will have access to faster and more precise diagnoses. For this reason, this journal will continue to value reports of local testing and adaptation and other forms of implementation science.

Alongside bench technique improvements are the quality management systems that will ensure that each patient sample is appropriately processed in time to influence care. Innovation is needed even here and this volume includes some interesting new initiatives. Coetzee et al.^9^ (this issue) demonstrate a simple mechanism for using laboratory data to measure performance, turn-around time in that specific instance, and Ismael et al.^10^ describe an external quality assessment programme for early infant HIV diagnosis.

To process laboratory specimens locally, and to the highest standards, it will often be necessary to upgrade or entirely refurbish existing laboratories or plant new ones. While this prospect may be seen as difficult or even daunting, the experiences of others, such as Beyanga et al.^11^ and Mtisi et al.^12^ (both this issue) are essential for replicating success and avoiding costly pitfalls. Beyanga et al.^11^ outline the path of a Tanzanian diagnostic laboratory to accreditation. In their description of the construction of a specialised pharmacology laboratory capable of performing pharmacokinetic analyses, Mtisi et al.^12^ outline the process from the stage of initiating public-private international partnerships, through human resource development to preparations for ISO 15189 accreditation. Ultimately, as Mtisi et al.’s article^12^ emphasises, quality must be an initial goal and not an afterthought. It is not sufficient for laboratories to claim to have performance capabilities; these must be assured. And while the majority of African diagnostic laboratories remain to be accredited, step-wise progression towards this goal is essential for ensuring diagnostic sufficiency in the near future.
